# A baseline structure inventory with critical attribution for the US and its territories

**DOI:** 10.1038/s41597-024-03219-x

**Published:** 2024-05-16

**Authors:** Hsiuhan Lexie Yang, Melanie Laverdiere, Taylor Hauser, Benjamin Swan, Erik Schmidt, Jessica Moehl, Andrew Reith, Daniel Adams, Bennett Morris, Jacob McKee, Matthew Whitehead, Mark Tuttle

**Affiliations:** Geospatial Science and Human Security Division, Oak Ridge National Laboratory, Oak Ridge, USA

**Keywords:** Geography, Natural hazards

## Abstract

Leveraging high performance computing, remote sensing, geographic data science, machine learning, and computer vision, Oak Ridge National Laboratory has partnered with Federal Emergency Management Agency (FEMA) to build a baseline structure inventory covering the US and its territories to support disaster preparedness, response, and recovery. The dataset contains more than 125 million structures with critical attribution, and is ready to be used by federal agencies, local government and first responders to accelerate on-the-ground response to disasters, further identify vulnerable areas, and develop strategies to enhance the resilience of critical structures and communities. Data can be freely and openly accessed through Figshare data repository, ESRI’s Living Atlas or FEMA’s Geodata platform.

## Background & Summary

In 2016, the United States experienced 32 major disasters and six emergency declarations involving floods. To effectively prepare for, respond to, and recover from disasters, spatially accurate data on critical infrastructure is essential. Precise location and building outlines provide the most accurate data for characterizing the impacts of hazards and serve response, recovery, and mitigation efforts, as well as those affected by the disaster. However, a comprehensive and usable open-source national database of building footprints does not currently exist. In this Data Descriptor, we present a complete workflow, built over a six-year period, for establishing the first comprehensive building inventory with critical attribution, such as address and structure use, to support disaster response in the United States. We call this the USA Structures database. This workflow leverages novel scientific and technological capabilities in the broad areas of geographic data science, socio-cultural characterization of population and landscape processes, machine learning, computer vision, and geocomputation at scale. Our workflow includes imagery curation and pre-processing, developing computer vision building extraction models for country-scale use, quality control and validation processes, and finally attaching several critical attributes derived from authoritative sources to the detected structures.

## Methods

**Fig. 1:**
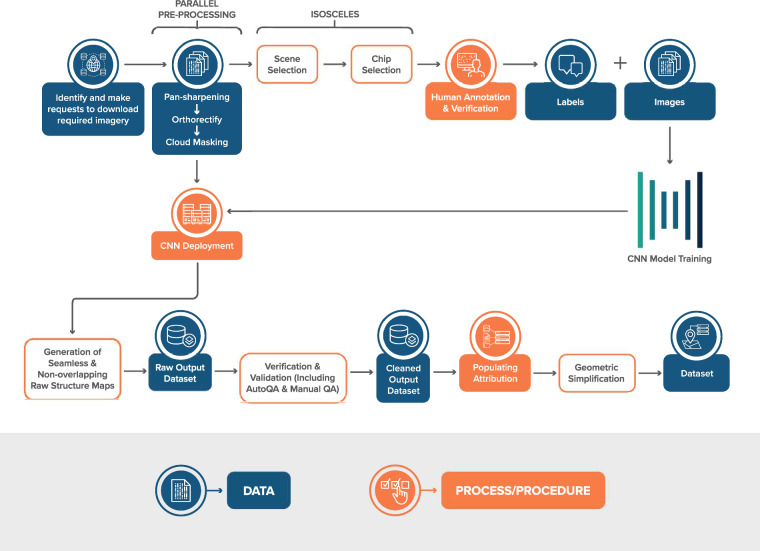
USA Structures workflow.

In recent years, several building outline datasets have become publicly available (e.g. Microsoft and Google building outlines^1,2^). However, these products lack building metadata and other critical attributions. In this data descriptor, we provide details of a proposed workflow for establishing a seamless structure inventory for the United States, aiming to not only provide the polygons of buildings (hereafter structures) but also to provide relevant metadata for structures and critical attributions to support disaster response, disaster preparedness. Further, the dataset can support stakeholders to identify vulnerable areas, and develop strategies to enhance the resilience of critical structures and communities. We discuss the details of each step of the workflow below, including **Imagery Curation and Pre-processing,** **Label Set Building,** **Convolutional Neural Network Training and Deployment,** **Verification and Validation,** **Adding Structure Attribution** and the **Geometric Simplification**. The overall workflow is illustrated in Fig. 1.

### Imagery curation and Pre-processing

Based on our preliminary country-scale building mapping efforts^3^, we demonstrated the possibility of mapping structures with high resolution (~1-meter ground sampling distance (GSD)) aerial images. We further identified the need to exploit higher resolution images, beyond 1-meter GSD, so that the outlines of detected structures, especially those with smaller buildings, are more discernible in overhead images and therefore detectable in machine learning based feature extraction. We exploited WorldView-02 and -03 imagery from Maxar and U.S. Department of Agriculture (USDA) National Agriculture Imagery Program (NAIP) aerial imagery from the United States Geological Survey (USGS). Since high spatial resolution and temporal currency are most relevant to the goal of creating a high quality and accurate building dataset, WorldView-02 and WorldView-03 served as the main imagery resources. In the event of Maxar coverage gaps or lack of favorable images due to imagery quality concerns or cloud cover, we used other available images with comparable spatial resolution such as QuickBird, GeoEye-1 or NAIP.

In order to use the full potential of satellite images and offer maximum flexibility to process the latest images when needed, we developed an in-house imagery pre-processing pipeline^4^ to perform pan-sharpening and orthorectification. Several imagery curation and selection criteria were used. Selected images from WorldView-02, WorldView-03, or other satellite sensors were prioritized based on the most recent image acquisition date, minimal cloud cover, and high spatial resolution between 30–70 cm.

In total, we processed ~90,000 images, approximately 1.2 PB with collection dates ranging from 2011–2021.

### Develop building extraction models using convolutional neural networks

Although building mapping with high resolution remote sensing images has been an active research for many decades, the major breakthrough in efficiency and performance was made when the researchers started leveraging convolutional neural networks (CNN) based approach since 2016^5–7^. One of the requirements to achieve this outstanding performance in object detection, image classification or semantic segmentation tasks is the availability of labelled data. Therefore, we first needed to compile a set of labelled data to support developing building extraction CNN models.

#### Data-driven sample selection for labelling

While leveraging existing high quality small-scale footprint data^3^ or noisy large-scale data, such as OpenStreetMaps, to generate labelled data might be a suitable solution, we have found that the quality and quantity of labelled samples plays a critical role in structure mapping results^8^. We took the rather costly and more time consuming approach of manually digitizing labelled training data to ensure a high quality machine learning output. In our previous work^3^, training a building extraction model using NAIP images did not encounter the model generalization issue resulted from variability of images, as NAIP imagery is fairly consistent in having low-off nadir viewing angles, time of data collections (i.e. leaf-on seasons) and radiometric characteristics across all states, with post-processing used to histogram balance each individual image. However, the high-resolution satellite imagery we have used exhibits greater variability in looking angle, sensor types, and time/date of imagery collection.

Since creating high-quality, manually labelled training data is costly in both time and money, we needed a way to efficiently select salient samples for labelling. This challenge was compounded by the need to capture multi-dimensional variability across very large image domains. To address this challenge, we developed a data-driven sample selection process and program, ISOSCELES^9^, which automates the process of image sampling through hierarchical unsupervised data clustering.

**Fig. 2:**
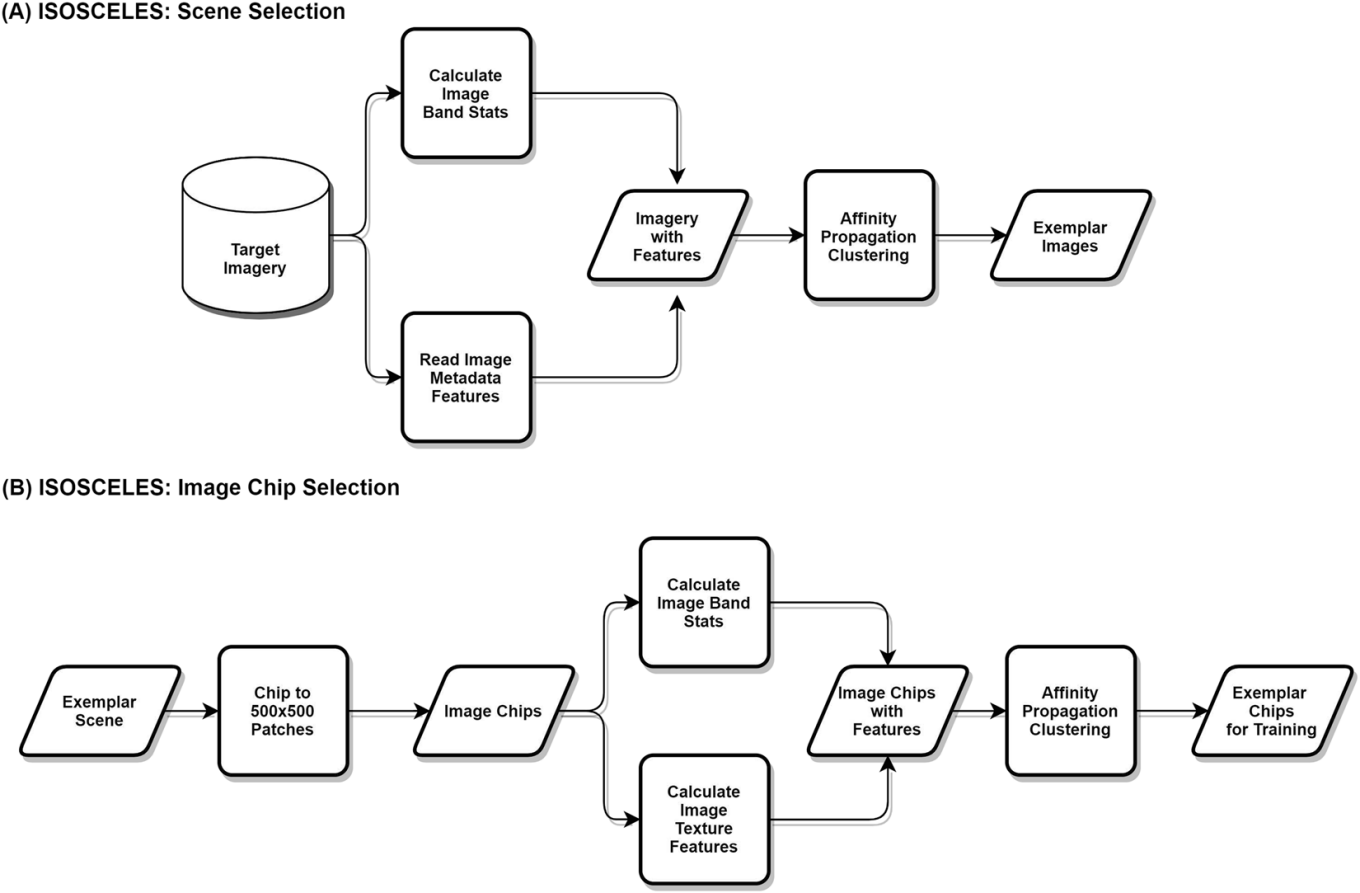
Data sampling process.(Adapted from^9^).

ISOSCELES operates on two scales, first selecting highly representative images from the full satellite image set (Fig. 2a), then selecting highly representative subsets (hereafter image chips) from those scenes that can then be labelled to create supervised training data (Fig. 2b). Thus, we can capture both between image variability in characteristics such as viewing and sun elevation and within image variability from such characteristics as building style and land use/land cover (LU/LC) contexts. We have been able to verify the efficacy in a large-scale experiment, detailed in^9^, which showed significant improvements in both precision and recall when using the ISOSCELES strategic sampling program compared to stratified random sampling.

**Fig. 3:**
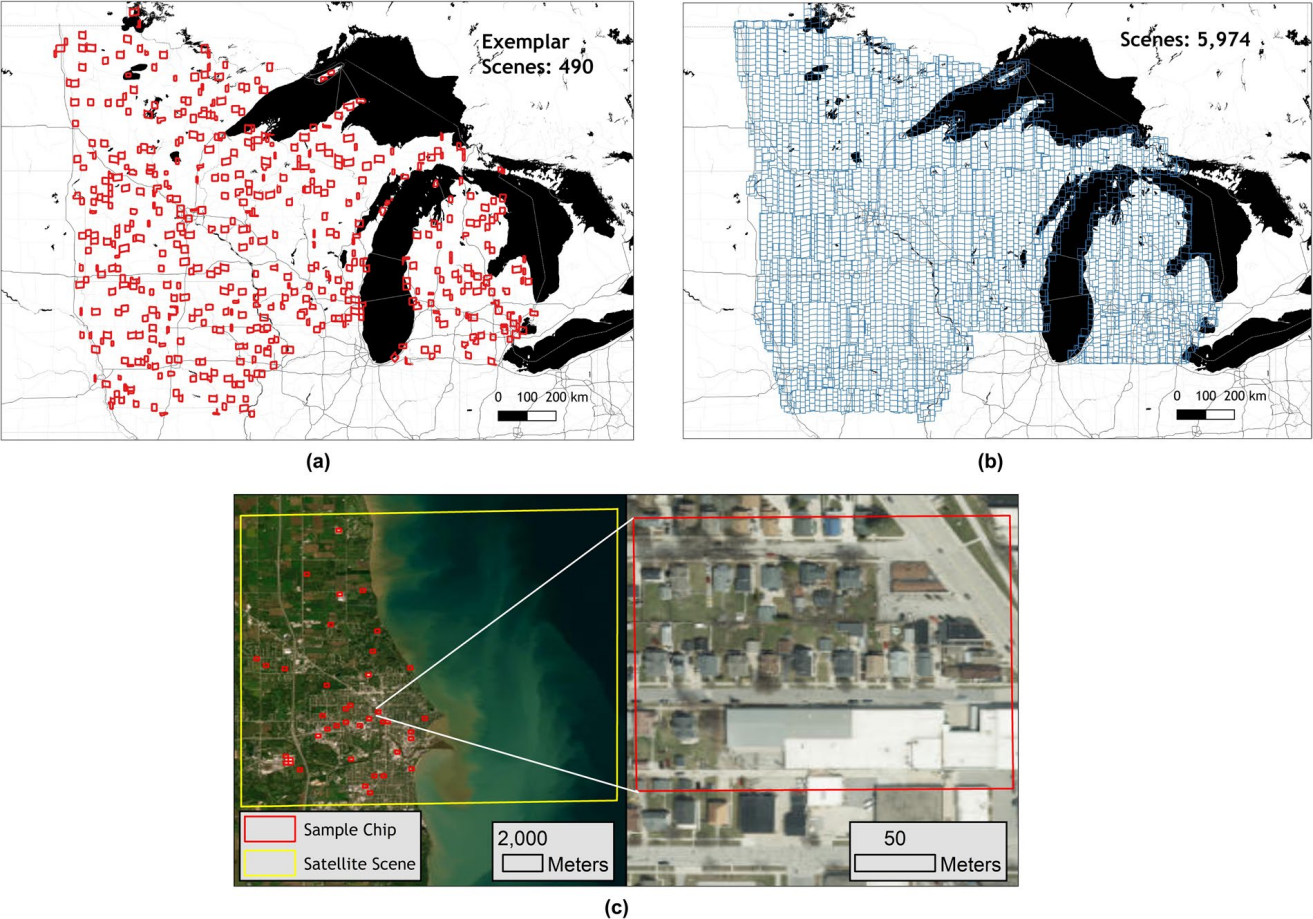
Example of ISOSCELES sampling for the Upper Midwest states. (**a**) Full set of non-overlapping source imagery used for Upper Midwest States building extraction. (**b**) Exemplar scenes selected in first stage of ISOSCELES sampling. (**c**) Exemplar scenes and exemplar sample selected at the second stage of ISOSCELES sampling.

We show the example of the resulting samples for the Upper Midwest states in Fig. 3, where Fig. 3a shows the original full set of images after the initial imagery curation step, Fig. 3b shows the resulting exemplar scenes after Fig. 2 step, and then Fig. 3c demonstrate the final selected sample image chip for manual labelling.

**Fig. 4:**
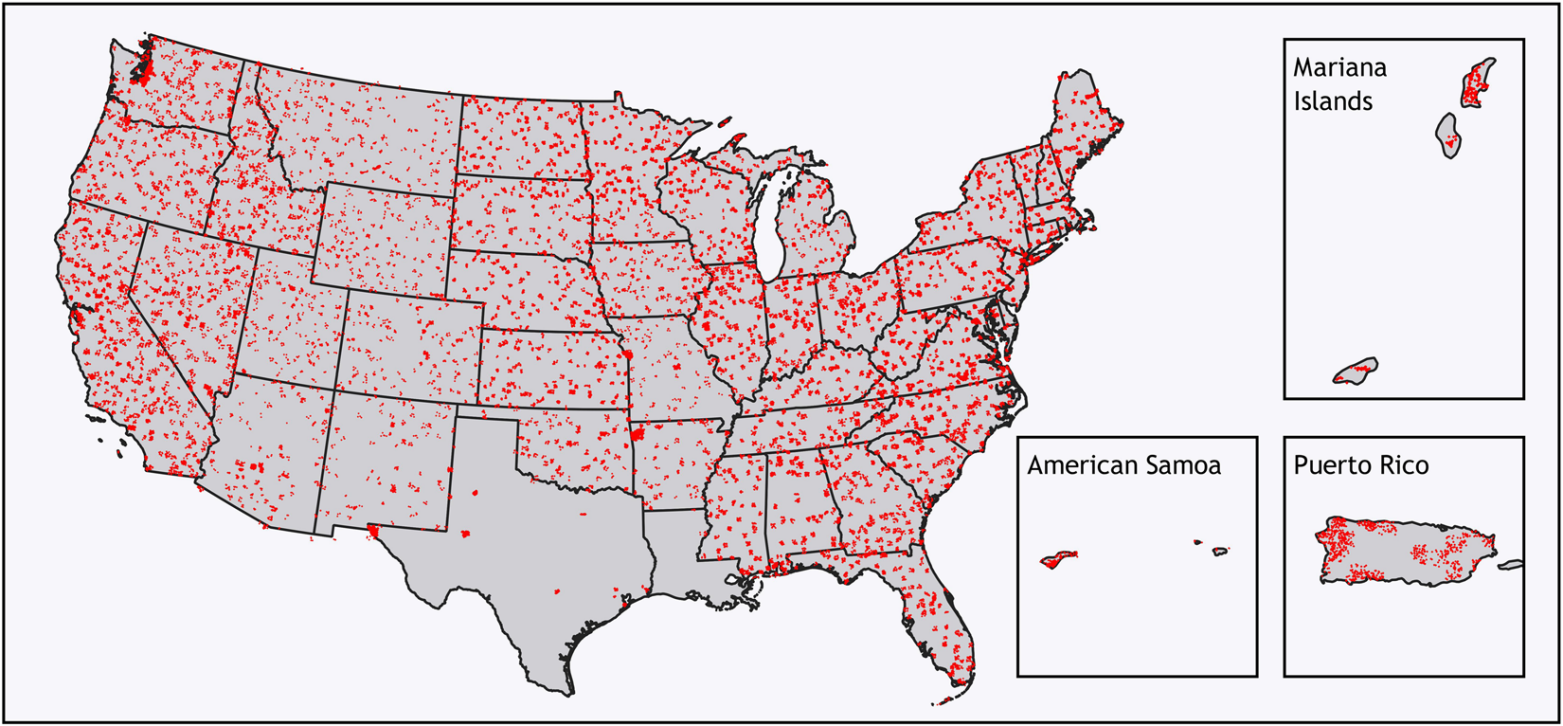
Spatial distribution of labelled samples across United States.

After selecting representative image samples based on the above data-driven sampling strategy, those samples were annotated by in-house GIS analysts with binary labels (structures and non-structures) based on a annotation guideline for keeping label consistency. Then, the signed-distance labels can be derived from the binary labels, as illustrated in^3^. We have 59,000 manually created training samples. The distribution of those samples are shown in Fig. 4.

#### Development of CNN models

We have developed and advanced several CNNs to extract structures from satellite imagery automatically. This task was framed as a binary semantic segmentation problem, where each pixel in the imagery will be classified as structure or non-structure. Starting with the basic CNN architecture described in^3^, we were continuously adding, benchmarking and advancing CNN architectures and feature learning modules to improve the quality of the structure extraction results. Various modules and loss functions were tested, including residual modules^10^, attention modules^11^, focal tversky loss^12^. The CNN architecture we used mostly is a U-Net^13^ based multi-task architecture with signed distance labels^3^, given both its consistent performance across different states and structure types and its computational efficiency in processing massive amount of imagery. The multi-task CNN has two heads; one aims to learn the completeness of extracted structures, which is guided by binary labels, while the other head learns precise boundaries of structures with the help of signed-distance labels.

The building extraction models were trained using standard stochastic gradient descent approach on batches of labelled image samples from each FEMA-defined region, as summarized in Table 1. The regions are listed chronologically based on when models were developed for each area during the course of this work. As more training data became available over time, model performance generally improved thanks to greater exposure to diverse examples during training.

**Table 1:** The metrics of CNN building extraction outputs.

States/Territories	Train/Val	Precision	Recall	F1-score
Texas, Louisiana V1	1,400/200	0.831	0.862	0.846
Arkansas, Missouri	1,300/200	0.871	0.860	0.865
Puerto Rico	858/66	0.930	0.920	0.925
Oklahoma	994/110	0.892	0.886	0.889
Arizona, New Mexico	2,129/150	0.917	0.917	0.917
Alabama, Mississippi	2,067/299	0.882	0.912	0.897
Georgia, South Carolina, Florida, North Carolina	4,983/946	0.842	0.909	0.874
Kentucky, Tennessee, Illinois, Indiana, Ohio	1,200/200	0.902	0.793	0.844
California, Nevada	6,505/735	0.913	0.910	0.912
Idaho, Oregon, Washington	6,213/500	0.946	0.829	0.884
Connecticut, Maine, Massachusetts, New Hampshire, Rhode Island, Vermont, Delaware, District of Columbia, Maryland, New Jersey, New York, Pennsylvania, Virginia and West Virginia	7,618/1,106	0.856	0.848	0.852
Iowa, Michigan, Minnesota, Wisconsin	5,258/1,421	0.907	0.869	0.887
Kansas, Nebraska, North Dakota and South Dakota	5,545/944	0.932	0.936	0.934
Colorado, Montana, Utah, Wyoming	2,428/815	0.922	0.914	0.918
Mariana Islands	391/60	0.840	0.816	0.828
American Samoa	395/56	0.934	0.893	0.913
Texas, Louisiana V2	1,400/200	0.916	0.935	0.926

Note: Alaska, Hawaii, Guam, and the Virgin Islands were all processed using pre-existing models or US base-models generated based on all samples available. No new labelled training data or models were created for these states/territories. There are two versions of structures for Texas and Louisiana. **Texas, Louisiana V2** are the metrics obtained for producing the current version of structures in this dataset.

The overarching model development strategy relied on transfer learning from a collection of pre-trained model for each region over time. The best base model was selected by comparing validation accuracy across multiple candidate pre-trained models, then fine-tuned using additional region-specific hand annotated training samples. After the new labelled data collected for the states of Idaho, Oregon, Washington were completed, we had accumulated enough training samples to produce several versions of generalizable pre-trained “US base-models”, which were obtained by iteratively re-training with all available labels (existing or newly created batches of samples for each sets of new states). By examining the performance metrics such as F1 score, precision, and recall, these US base-models consistently outperformed previous regional models when exposed to validation samples from new states and thus were used as our base model for fine-tuning on all subsequent project areas. By combining ISOSCELES sampling and the generalizable US pre-trained models, we ensured that diverse labelled samples from new states were included in the fine-tuning process. This enabled the CNN to learn better while reducing labeling efforts and accelerating the production of raw structure extraction results.

All models were trained for 100 epochs, with the best checkpoint chosen for deployment based on highest validation accuracy. The F1-score was usually the deciding metric for choosing a production model. However, in areas such as Kentucky, Tennessee, Illinois, Indiana, and Ohio, there’s an exception due to persistent issues of high false positive rates caused by image quality problems. In these cases, models with the highest precision are preferred. The selected optimized model was then used for running inference on all of the images covering the entire target region.

### Quality checking and validation

#### Automatic QA/QC

Quality assurance and check is a crucial step in transforming CNN feature extraction output into operationally capable datasets for disaster response. The size of the raw structure detections are beyond what analysts can review manually in a timely fashion, i.e. millions of polygons. We developed an automatic verification and validation process based on a binary supervised classification machine learning algorithm. In this Verification and Validation Model (VVM) there are 22 features derived from raw detections used to distinguish the false positives and true positives. There are four general types of features calculated for the verification: geometric, engineered (derived by two or more geometric features), ancillary (additional data sets to generate), and contextual (derived from the geometry and the spatial and scale relationship of nearby geometries). We trained four different classification and regression trees machine learning algorithms and evaluated the performance by F-1 scores. Then we selected the highest performing algorithm to be the classification algorithm for the VVM. Development of the VVM is described in more detail in the QA/QC results section. The steps for the automated QA/QC are as follows:

1. First, we remove raw structure extractions outside the area of interest (AOI) boundary. Although the dataset was developed state by state, the image scenes used for structure extractions often extended beyond the official AOI defined by the 2020 census state boundaries, in order to maximize coverage.
2. Secondly, we generate VVM features required to evaluate the remaining structure extractions. There are 22 different measures of morphology calculated for each raw predicted structure feature.
3. Then, we use VVM to analyze the morphology of each structure and assign a true positive probability. This probability ranges from 0 to 100, with 100 indicating high confidence that the extracted structure is indeed a true structure, and 0 indicating high confidence that it is a false positive.
4. Next, we remove raw extracted structures that do not meet the following two thresholds: 1) the area of the structure must be greater than 450 square feet, which is approximately the size of a single-wide mobile home, and 2) the VVM true positive probability must be 50 or higher.
5. Finally, the final outcomes are overlaid on top of the raw structure output to identify areas where commission errors are occurring. This process helps analysts identify problematic areas that require further evaluation in the manual QA/QC process, or the need to improve VVM.

#### Manually review and identify gaps

**Fig. 5:**
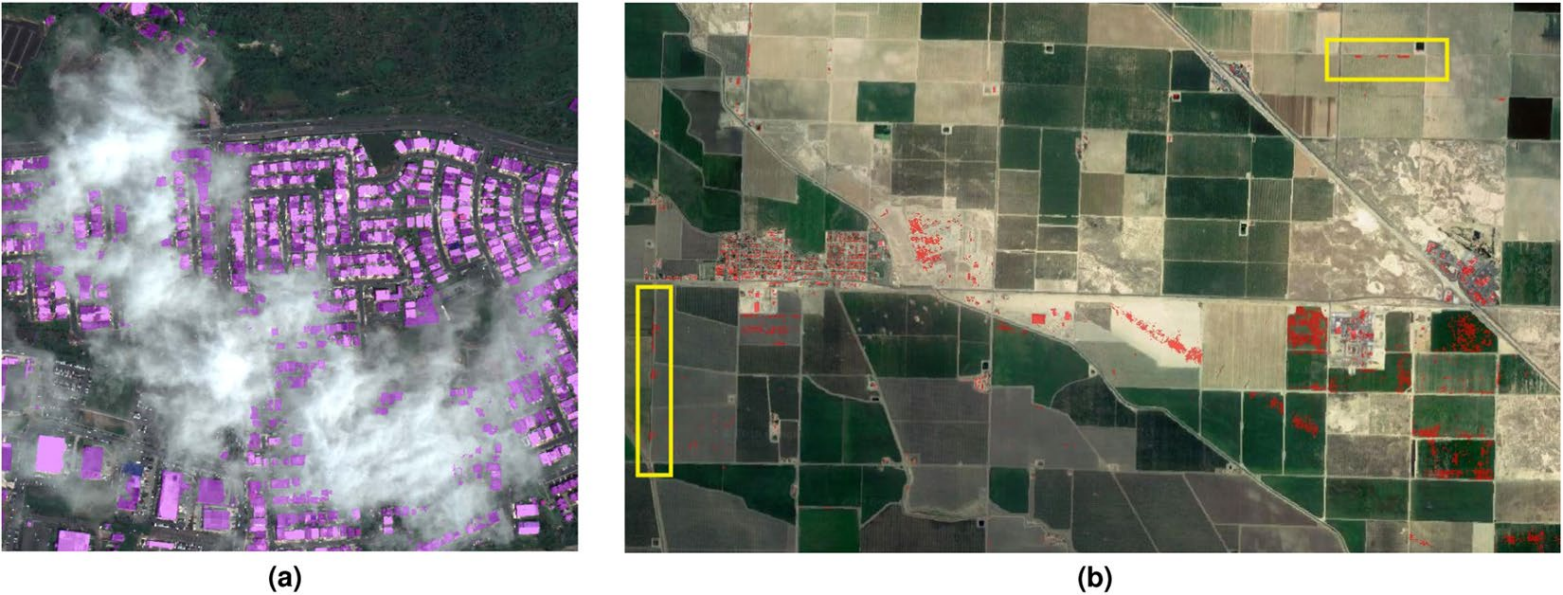
Example of unfavorable structure extraction outcomes due to poor image quality or overly complex patterns that are not able to be removed during automatic QA/QC. (**a**) Raw structure extraction results colored in purple. Note the omissions due to the clouds. (recreated from^4^). (**b**) Additional false positives that require manual QA/QC. The false positives in the yellow boxes are particular difficult to filter out by VVM.

After the automatic QA/QC process, analysts further examined the data layers. The inspection process involved reviewing and confirming the results of the automatic QA/QC. It also included identifying areas where poor image quality (clouds, haze, etc.) or acquisition characteristics (high view angle, time of day, etc.) resulted in undesirable outcomes. These outcomes included poor structure extraction results, which led to overly complex geometries for automatic QA/QC or omissions. We then replaced those with other sources such as LiDAR-derived structures (where available) and/or structures derived from lower-resolution NAIP imagery^3^, or manually removed those incorrect structure extractions. We give two examples to illustrate such scenarios in Fig. 5.

### Building attribution

After mapping the structures from high-resolution satellite images, and automatic and manual quality assurance processes were completed, we further enriched the structure inventory with building attribution by leveraging several authoritative data sources. Attribution of the buildings provides greater context and enables broader applications. A list of attributes developed for this database is shown in Table 2. The standard attribution for a subset of fields is populated by conflating extracted structures with varying source data (e.g. Census Tiger 2010 data^14^, source imagery metadata, and internal production information) via a large scale spatial join. Other standard fields are populated based on a structures geospatial descriptors, such as area and coordinates. There are two categories of attributions require carefully designed workflow to process various source data. We layout the details and data conflation steps in the following:

**Table 2:** The attribution schema for the USA Structure data set.

Field	Set	Description	Source
OBJECTID	Standard	Esri default data set specific unique ID	Esri
SHAPE	Standard	Esri default geometry field	Esri
BUILD_ID	Standard	State specific Unique ID	ORNL
OCC_CLS	Standard	Top level building occupancy class	HIFLD, LB, Census, or ORNL
PRIM_OCC	Standard	Primary descriptor for a a building’s usage	HIFLD, LB, Census, or ORNL
SEC_OCC	Unpopulated	Range of units within Multi Family Dwellings	No source Identified
PROP_ADDR	Standard	Primary street address	NAD
PROP_CITY	Standard	City name	NAD
PROP_ST	Standard	State name	Census Tiger
PROP_ZIP	Standard	Zip code	NAD
OUTBLDG	Unpopulated	Non-primary structure	No source Identified
HEIGHT	Standard	Measure of height in meters	ORNL
SQMETERS	Standard	Two-dimensional area of the building in square meters	ORNL
SQFEET	Standard	Two-dimensional area of the building in square feet	ORNL
H_ADJ_ELEV	Unpopulated	Highest elevation in meters of adjacent ground	No source Identified
L_ADJ_ELEV	Unpopulated	Lowest elevation in meters of adjacent ground	No source Identified
FIPS	Standard	US County Federal Information Processing Standards Code	Census Tiger
CENSUSCODE	Standard	Census tract identifier	Census Tiger
PROD_DATE	Standard	Date that structure was captured	ORNL
SOURCE	Standard	Name of organization that created the structure	ORNL, NGA, or FEMA
USNG	Standard	United States Nation Grid Coordinate at meter resolution	ORNL
LONGITUDE	Standard	Centroid longitude in millionths of decimal degrees	ORNL
LATITUDE	Standard	Centroid latitude of structure in millionths of decimal degrees	ORNL
IMAGE_NAME	Standard	Catalog ID or image name used to capture structure	Maxar, USGS, or NGA
IMAGE_DATE	Standard	Date of image acquisition	Maxar, USGS, or NGA
VAL_METHOD	Standard	Methodology of validation	ORNL
REMARKS	Standard	Additional Comments	ORNL or HIFLD
UUID	Standard	Universally unique identifier	ORNL
SHAPE_LENGTH	Standard	Esri default perimeter length of structure	Esri
SHAPE_AREA	Standard	Esri default area of structure	Esri

#### Structure occupancy type classification

The use of a structure is a critical attribute for a wide variety of applications, for example, emergency response, population modeling, and risk assessments. To improve utility for emergency response, we populated two attributes: (1) building occupancy type class, **OCC_CLS** in the Tables 2, and (2) the primary descriptor for a building’s usage for each top level building, **PRIM_OCC** in the Table 2. The categories were defined largely based on the HAZUS definition^15^.These two attributes were derived with a customized geospatial data conflation workflow that ingests several authoritative data sources, then filters and selects data layers, ranks them, preform spatial joining and final apply occupancy type attributes for a given structure by spatial conflation. The authoritative data sources we leveraged in this work are introduced below:

- 57 Homeland Infrastructure Foundation Level Data (HIFLD)^16^ data layers
  HIFLD is a set of foundational datasets related to domestic national security and emergency response. This collection of national geospatial datasets focus on mapping the nation’s critical infrastructure and include standardization of schemas and attribution. In this work, we needed to map and aggregated HIFLD layers to a special USA Structure schema that is most informative to meet the needs of disaster response. The mapping was conducted by team members who heuristically mapped each layer to the most appropriate classification in the schema. The mapping is shown in Table 3.

**Table 3:** Mapping HIFLD to PRIM_OCC and OCC_CLS.

HIFLD Layer Name	OCC_CLS	PRIM_OCC
Agricultural Minerals Operations	Industrial	Food/Drugs/Chemicals
All Places of Worship	Assembly	Religious
Bio Diesel Plants	Industrial	Food/Drugs/Chemicals
Child Care Centers	Education	Other Educational Buildings
Colleges and Universities	Education	Colleges/Universities
Colleges and Universities campuses	Education	Colleges/Universities
Convention Centers and State Fairgrounds	Assembly	Convention Center
Courthouses	Government	General Services
DOD Sites Boundaries Public	Government	Non-Civilian Structures
DOD Sites Points Public	Government	Non-Civilian Structures
EPA Emergency Response Facilities	Government	General Services
Ethanol plants	Industrial	Food/Drugs/Chemicals
Ethanol trans loading facilities	Commercial	Wholesale Trade
FDIC Insured Banks	Commercial	Banks
Fedex Facilities	Commercial	Wholesale Trade
Ferrous Metal Mines	Industrial	Metals/Minerals Processing
Ferrous Metal Process Plants	Industrial	Metals/Minerals Processing
Fire Station	Government	Emergency Response
FDA Office Facilities	Government	General Services
Fortune 500 Corporate Headquarters	Commercial	Professional/Technical Services
General Manufacturing Facilities	Industrial	Light
Government Financial Processing Centers	Government	General Services
Governors Mansions	Government	General Services
Hospitals	Commercial	Hospital
Liquified Natural Gas Import Exports and Terminals	Commercial	Wholesale Trade
Local Emergency Operations Centers	Government	Emergency Response
Local Law Enforcement Locations	Government	Emergency Response
Major Sport Venues	Assembly	Indoor Arena
Major State Government Buildings	Government	General Services
Mines and Mineral Resources	Industrial	Metals/Minerals Processing
Miscellaneous Industrial Mineral Operations	Industrial	Metals/Minerals Processing
Natural Gas Processing Plants	Industrial	Food/Drugs/Chemicals
NCUA Insured Credit Unions	Commercial	Banks
Nonferrous Metal Mines	Industrial	Metals/Minerals Processing
Nonferrous Metal Processing Plants	Industrial	Metals/Minerals Processing
Nursing Homes	Residential	Nursing Home
Oil and Natural Gas Platforms	Industrial	Food/Drugs/Chemicals
Oil Refinery Polygon	Industrial	Food/Drugs/Chemicals
Oil Refineries	Industrial	Food/Drugs/Chemicals
Petroleum Ports	Commercial	Wholesale Trade
Petroleum Terminals	Commercial	Wholesale Trade
Pumping Stations	Commercial	Wholesale Trade
Power Plants	Utility and Misc	Energy Control Monitoring
Prison Boundaries	Residential	Institutional Dormitory
Private Non-Retail Shipping Facilities	Commercial	Wholesale Trade
Private Schools	Education	Pre-K - 12 Schools
Public Health Departments	Government	General Services
Public Refrigerated Warehouses	Commercial	Wholesale Trade
Public Schools	Education	Pre-K - 12 Schools
Sand and Gravel Operations	Industrial	Metals/Minerals Processing
Solid Waste Landfill Facilities	Utility and Misc	Ground
State Capitol Buildings	Government	General Services
Supplemental Colleges	Education	Colleges/Universities
Truck Driving Schools	Education	Other Educational Buildings
UPS Facilities	Government	General Services
Urgent Care Facilities	Commercial	Medical Office/Clinic
Veterans Health Administration Medical Facilities	Government	General Services
- Lightbox smart parcels^17^
  The Lightbox smart parcels are provided through HIFLD Licensed via a data agreement for federal use cases^18,19^.
- US Census housing unit data^20^
  The US Census Bureau provided the team with a special tabulation of housing unit percentages at the block level from the 2010 census^14^. This data layer is comprised of percentages of houses that are either Single-Family, Multi-Family, Manufactured or Other.
- Department of Housing and Urban Development (HUD)^21^
  From US Department of Housing and Urban Development (HUD) open data platform, we obtained points data represent addresses of properties that are assisted or insured through HUD^21,22^.
- OpenStreetMap (OSM)^23^
  We downloaded a polygonal dataset from OpenStreetMap (OSM) that is a selection of all the polygons with the key “aeroway”^24^. This key is used for many features relating to airport structures.
- Federal Aviation Administration (FAA) layers^25^

This is a polygonal dataset that designates airport runways from the US FAA’s open data platform. These polygons represent takeoff and landing areas^25^.

The first three sources were used to determine the vast majority of structures’ occupancy type attribution. The last three were used to determine “Multi-Family Dwelling” (from HUD data source) and “Aviation” (from OSM and FAA layers) in the **PRIM_OCC** attribute. In addition, for some geographies, namely Northern Marianas and Puerto Rico, we also obtained local parcel coverage to be the input data layers as the part of structure occupancy type classification workflow.

The overall workflow of assigning a building **OCC_CLS** to the structure polygon is a series of spatial join and intersection. Considering the data reliability and accuracy, the order of the data layers in this spatial join and intersection is HIFLD, Lightbox smart parcels, and Census housing unit. In general, the steps of classifying an occupancy type of a given structure is: 1) If a structure intersects with HIFLD layer, then the structure occupancy type will be determined by the type or theme of the HIFLD layer. 2) Any structure that does not intersect HIFLD data layers will be then checked if it intersects with LightBox smart parcels. If the structure falls within a parcel that has a land use type appropriate for the USA Structure schema, then the parcel is used to determine the occupancy type. 3) If no occupancy type has been determined by this step, the next source used is the Census housing unit data. 4) Lastly, if a structure remains unclassified, a machine learning based residential binary classifier named **ResType**, which exploits the same set of features derived during the automated QA/QC process, provides a final determination of the structures occupancy type.

The goal of using this classifier is to assign those remaining unlabelled structures as residential or non-residential. The machine learning classifier was created in a supervised manner, where the training labels (i.e. residential or non-residential) came from an aggregation of land use codes in the parcel data set. To train the classifier on the most representative or typical records, thereby reducing the influence of anomalies, a one-class support vector machine with a *v* value of 0.35 was employed to filter the training data. In addition to this, to mitigate the negative impact of imbalanced label set during **ResType** training, we performed undersampling on the larger class. For example, in FEMA Region 1 (Connecticut, Maine, Massachusetts, New Hampshire, Rhode Island and Vermont), we randomly sampled 275,662 samples from the total 2,443,319 residential label samples to match the total of nonresidential labels. If the structure remains unlabelled after consulting aggregated HIFLD data, aggregated Lightbox parcel data, HUD, then it is evaluated using the residential classifier and assigned either residential or unclassified (implied non-residential). Thus, all the structures will be assigned an occupancy type.

#### Addresses

As the most common means of identifying structures and referencing their locations, street addresses are a key component for linking structure data to other datasets, a common effort for FEMA when responding to an emergency event. The address data included in USA Structures were derived from publicly-available and open sourced data. While we identified some open state sources, the primary source for addresses was the National Address Database (NAD), a U.S. Department of Transportation (DOT)-led effort to collate and distribute a standardized geospatial dataset of addresses in the U.S.^26^. As of March 2023, DOT has partnered with state and/or local governments in forty states to deliver address data covering most of the U.S., though some partners have yet to provide data. In those areas without NAD coverage, we identified state sources where available; however, some states either have no open address data or do not make them available to the public, so gaps in address information are present in some areas of USA Structures. The address data is here referenced as comprising three components: street address, city, and postal code, which mapped to the **PROP_ADDR,** **PROP_CITY**, and **PROP_ZIP** fields, respectively.

Given overlapping geographic coverage and varying completeness of these sources, we built a pipeline to measure the completeness and validity of each record in our sources to ensure that, for each structure, we selected the best available address from all available sources. Street addresses, for example, were considered valid if they possessed at least three components: an address number, street name, and street type (road, street, lane, etc.). To measure completeness, each address component was weighted based on its specificity and importance to the address overall, whereby street address was prioritized over city, and city over postal code. If a component was missing or deemed invalid, that was captured in the address record’s rank as shown in Table 4. For example, a record with a valid street address and no other information is prioritized over a record with only city and postal code information. In this way, we ensured that the best, most complete addresses were prioritized for conflation with our structure geometry.

**Table 4:** Address data ranking and scores table.

Rank	Complete/Valid Fields	Example
1	Street address, Unit number, City, Postal code, State	101 Smith Rd, Unit B, Pleasantville, 47220, IN
2	Street address, City, Postal code, State	101 Smith Rd, Pleasantville, 47220, IN
3	Street address, City, Postal code	101 Smith Rd, Pleasantville, 47220
4	Street address, City	101 Smith Rd, Pleasantville
5	Street address, Postal code	101 Smith Rd, 47220
6	Street address	101 Smith Rd
7	City, Postal code	Pleasantville, 47220
8	City	Pleasantville
9	Postal Code	47220
10	No valid fields	101 Rd, P8, 477

We use the known characteristics of the address data to determine the best geolocation mapping for selection. Some address points are on entity, or rooftop, therefore we can assume that if an address point intersects a structure, that address can be assigned to that structure. If more than one address point meets this criterion, we leverage the ranking methodology outlined above to select the best address for conflation. Intersection can also be used in the opposite direction if the address source is polygonal, such as is the case with Florida’s parcel dataset. If a structure centroid intersects a parcel, we assume the address can be assigned to this structure.

After assigning structure addresses based on intersections, we select the structures that did not get an address from intersection or that have a rank higher than 6. We then calculate the nearest addresses by intersecting the addresses and structures with parcels. The nearest, best ranked address is selected for conflation. A structure can only be assigned an address if it is within the same parcel and also within 350 feet of address point. Through testing and observations, we found this process to yield the best results, but there are many limitations, which we outline in a later section.

### Geometric simplification

Geometric simplification (or shape regularization in certain literature) is the process of removing incidental vertices from polygons while not changing the overall form of the geometry. This process has many benefits to the user. First, the geometries on average have over 90% of their vertices removed which makes them easier to store. This reduces the overall data in terms of storage requirements. The second advantage of geometric simplification is increased rendering speed with most GIS software systems as the vertices will be reduced significantly. The last benefit is that the shape regularized structures conform to geometric rules, such as parallelism and perpendicularity, the resulting output is often more visually appealing and useful to applications. We used ArcGIS proprietary building footprint regularization module^27^ to accomplish this process. The parameters were set based on two underlying factors: Geometry quality and computational expense. Two parameters had significant impact on both factors, Tolerance and Precision. Tolerance is the maximum distance a footprint can deviate from its original position during geometric simplification. Precision determines the resolution of spatial grid used by the simplification process. Precision had the greatest affect on both quality and computation time. We observed a near exponential increase in computation for more precise geometries.

## Data Records

The dataset is available through Figshare^28^. This is also a mirrored dataset that was available in 2023 through the link to FEMA Geospatial Resource Center https://disasters.geoplatform.gov/USA_Structures/. Since there may be future updates to this dataset, we recommend citing the dataset using the above DOIs to accurately reflect the data version described in this Data Descriptor. The specific schema used for USA Structures was determined by FEMA for use in the broader emergency management community. A description of each polygonal structure and its associated attribution are listed in Table 2. The **OCC_CLS** and **PRIM_OCC** are generated through the occupancy type classification workflow described above. The **PROP_ADDR,** **PROP_CITY,** **PROP_ST**, and **PROP_ZIP** are produced during the address conflation process detailed previously. **HEIGHT** is populated if **SOURCE** of the structure is from in-house National Geospatial-Intelligence Agency (NGA) 133 cities data holdings. This data layer was produced with LiDAR, and was provided to our team as post-processed structure polygons with associated mean heights pre-populated. **PROD_DATE** indicates the date the post-processed polygonal building features were created. If the images were processed through the in-house image pre-processing pipeline, then the the catalog ID generated from imagery vendor and date of images will be documented in the **IMAGE_NAME** and **IMAGE_DATE**. The **VAL_METHOD** denotes if a given structure is validated manually, automatically via VVM or not at all. The Universally Unique Identifier^29^, **UUID**, is a unique 128-bit string in ‘{8-4-4-4-12}’ format for future tracking status of individual structures. This unique identifier was selected as opposed to another popular identifier, the Unique Building Identifier (UBID), in order to ensure unique buildings are assigned a unique identifier, irrespective of location. The UBID assigns a value based on geographic location. This is problematic in instances where a building is destroyed/demolished and a new structure takes its place. With UBID, these two unique structures would have the same identifier. With UUID, they are two separate designations. Lastly, the **REMARKS** field is currently only populated to designate between private and public hospitals. This was specifically requested by FEMA since disaster relief efforts may vary for public versus privately owned hospitals.

The values for other fields were automatically populated in ArcGIS (**OBJECTID,** **SHAPE_LENGTH** and **SHAPE_AREA**), or geometric characteristics calculated based on the polygonal structure (**SQMETERS,** **SQFEET**), or the locations (**USNG,** **LONGITUDE** and **LATITUDE**) of a given structure’s centroid, or extracted from auxiliary data sources (**FIPS,** **CENSUSCODE**).

There are other fields that are currently not populated (**SEC_OCC,** **OUTBLDG,** **H_ADJ_ELEV,** **L_ADJ_ELEV**), however, they might be updated in the future as ancillary data becomes available or other modeling techniques are developed.

## Technical Validation

### Validation results of building extraction with CNN

We divided the United States into sixteen regions based on a combination of FEMA’s desired delivery schedule (region order) as well as maximizing model suitability to combine states/territories with similar characteristics (region grouping). We then extracted raw structures from high resolution remote sensing imagery. We leveraged three different CNN architectures, which were evolved and improved based on the observations made at the CNN model outputs for each region. We reported the number of training and validation samples, precision, recall, and F1-scores calculated based on validation samples for each model in Table 1. The definitions of precision, recall, and F1-scores are given in the following:

1 $${\rm{precision}}=\frac{{\rm{TP}}}{{\rm{TP}}+{\rm{FP}}}\quad \quad {\rm{recall}}=\frac{{\rm{TP}}}{{\rm{TP}}+{\rm{FN}}}\quad \quad {\rm{F1}}-{\rm{score}}=\frac{2\cdot {\rm{precision}}\cdot {\rm{recall}}}{{\rm{precision}}+{\rm{recall}}}$$

where **TP** denotes true positives (i.e., correctly extracted structure pixels), **FP** denotes false positives (i.e., pixels mislabelled as structures), **TN** denotes true negatives (i.e., correctly identified non-structure pixels), and **FN** denotes false negatives (i.e., pixels incorrectly classified as non-structure by models or missed structure pixels as compared to the ground truth labels).

We used the validation samples to determine the convergence of the semantic segmentation CNN model training as well as the criteria to determine the quality of the outputs from CNN. Note that current version of structures for Texas and Louisiana is a result of fine-tuning the US-base model with all available labelled samples for these two states. Original structure dataset for these two states was produced as the first deployment of the CNN workflow, therefore the quality was less favorable due to the lack of training samples (see the sample distribution for Fig. 4 and exploratory nature of the pilot study states. After the development of ISOSCELES and the accumulated labelled images for CNN model training, we had seen significant improvements on the structure extraction results, as demonstrated in a much higher F1-score for **Texas, Louisiana V2**. Since these two states are prone to natural disasters, we updated the structure database to support FEMA’s disaster response with more accurate information.

### QA/QC Results

The training data for the VVM was developed by overlaying the structure detections with locally developed building footprints^30–34^. Any detection where the centroid of the detection intersected a building footprint was labelled as a true positive. The remaining detections within the training areas were the labelled as a **true positive** or **false positive** in a formal review process. We also paid attention to maintain class balance composition, by randomly sampling the larger class so that the number of samples will be equal to the minority class.

**Fig. 6:**
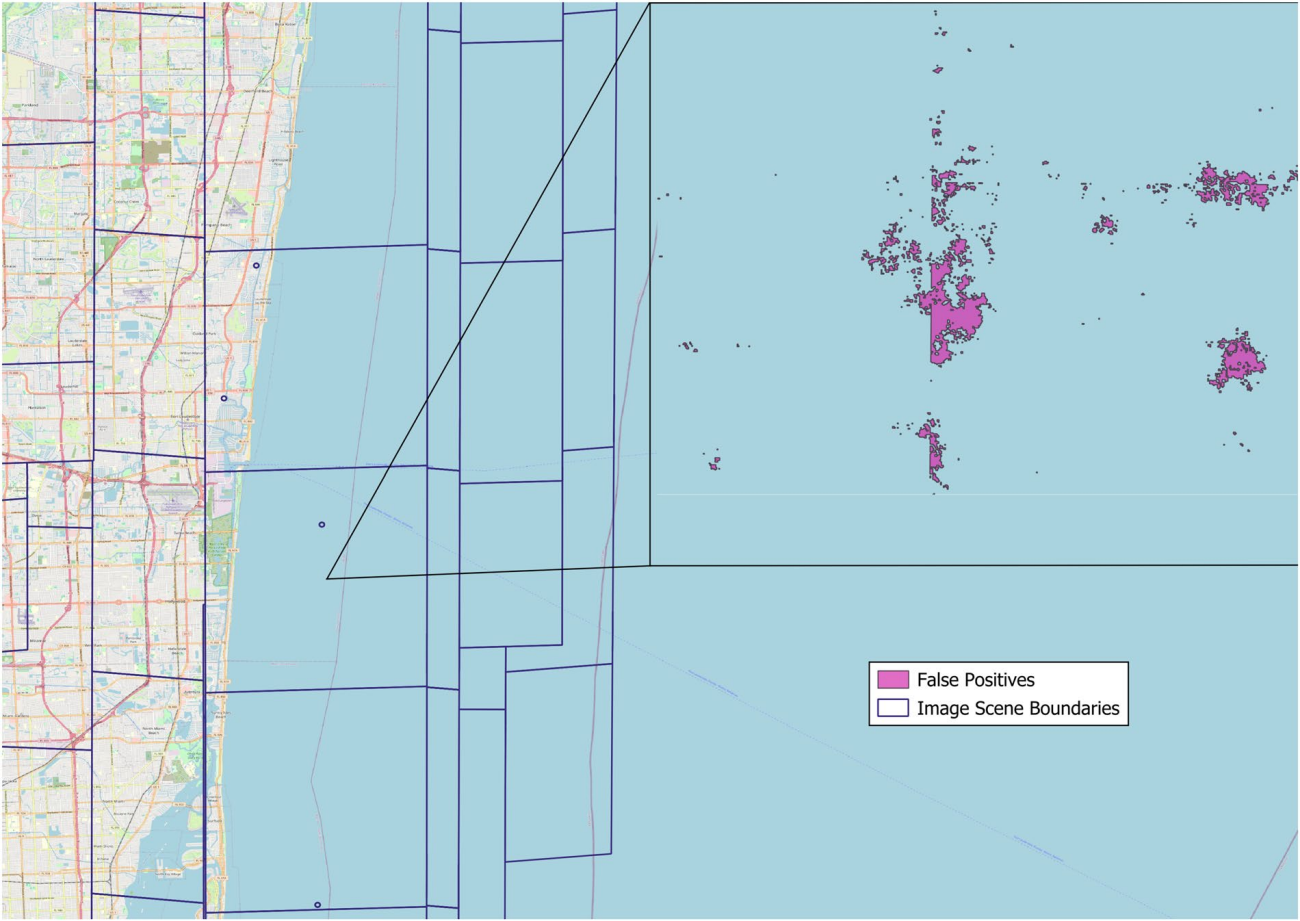
Kampville sample area, Validation of the VVM.

The results of the VVM inside the training areas had an overall accuracy of 94%. The model was trained on a random sampling of 66% of the data and tested against the remaining 33%^35^. The VVM maintains high performance and removes the vast majority of false positives while contributing very little to omission, e.g. removing a valid detection. More promising, are the results of the VVM outside the training area. The VVM was observed performing close to 99% overall accuracy in most areas as Fig. 6 demonstrates. The geometries are colored by their true positive probability, the output of the VVM. The lower the true positive probability the more confident the VVM is that the detection is a false positive (red). The higher the true positive probability the more confident the VVM is that the detection is a True positive (blue). Any detection with a true positive probability of 50% or greater was kept and assumed valid.

**Fig. 7:**
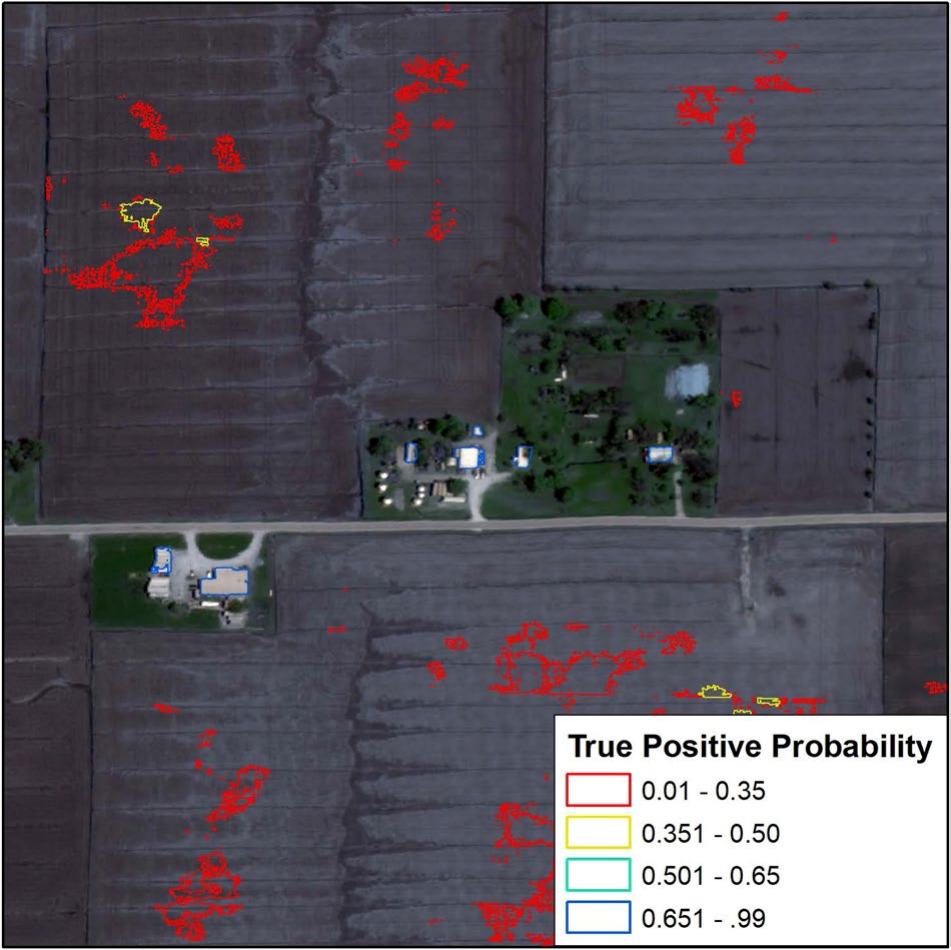
False positives (colored in purple in the left) over water bodies (ocean in this example).

We further provided the results of after applying VVM for each state in Table 5, where the **Raw Structure Count** indicates the number of feature counts from the raw building extraction results from CNN and the **Final Structure Count** indicates the the number of feature counts after applying VVM. We have observed that the majority of feature removal comes from two types of extraction results. The first type consists of small-sized raw results from CNN. This is because the area threshold (>450 square feet) is one of the criteria used to determine whether a given extracted structure should be included in the final dataset. Additionally, we have noticed that the geometric simplification process also generates some small artifacts, resulting in false positives. The second source of false positives that were frequently removed by VVM is the CNN outputs over large water body areas, as shown in Fig. 7. The images we used for CNN inference often extend to areas where there are no buildings, such as the ocean, rivers, and lakes. Many false positives are generated over water in dense clusters, with many of them being single pixel extractions. We have found that this type of false positives tends to persist, especially when imagery over water bodies is captured in windy environments where white cap waves are present.

**Table 5:** Number of extracted structures before and after VVM was applied.

State	Raw Structure Count	Final Structure Count	State	Raw Structure Count	Final Structure Count
Texas V2	24,978,385	11,597,857	Oregon	4,065,968	1,658,885
Louisiana V2	6,029,210	2,305,472	Iowa	4,436,185	2,114,520
Arkansas	3,852,879	2,489,884	Michigan	10,210,655	4,782,958
Missouri	6,231,759	1,527,560	Minnesota	6,376,765	2,801,654
Oklahoma	5,249,587	2,323,936	Wisconsin	6,237,633	3,039,604
Arizona	7,221,458	2,724,064	Virgin Islands	106,208	40,726
New Mexico	4,050,696	986,505	Kansas	3,321,184	1,600,218
Alabama	6,487,764	2,489,884	Nebraska	2,456,909	1,178,532
Mississippi	4,466,266	1,527,560	North Dakota	1,660,378	572,242
Guam	94,297	42,663	South Dakota	1,624,084	628,750
Hawaii	1,118,227	327,070	Tennessee	9,870,946	3,122,388
Puerto Rico	2,131,064	1,142,054	California	36,590,351	9,946,076
Georgia	11,106,805	3,757,825	Nevada	9,318,079	837,251
South Carolina	6,987,384	2,286,581	Idaho	2,489,660	853,335
Florida	21,448,424	6,645,067	Washington	7,315,968	2,780,681
North Carolina	13,255,310	4,650,575	Oregon	4,065,968	1,658,885
Illinois	31,194,534	4,639,278	Connecticut	2,185,889	1,131,222
Indiana	14,990,327	3,287,119	District of Columbia	108,040	58,061
Kentucky	10,269,496	2,418,871	Delaware	806,335	371,915
Ohio	35,585,663	5,496,516	Maine	2,172,324	761,802
Massachusetts	4,205,509	2,057,472	Maryland	3,087,898	1,658,164
New Hampshire	1,327,550	558,369	American Samoa	26,437	13,412
New Jersey	5,767,980	2,467,395	Northern Mariana Is.	45,118	12,572
New York	11,552,448	4,847,135	Colorado	4,594,455	2,174,948
Pennsylvania	11,017,503	4,837,949	Montana	2,113,769	767,753
Rhode Island	649,308	353,194	Utah	2,569,454	1,101,597
Vermont	935,347	357,733	Wyoming	1,268,169	385,465
Virginia	6,551,228	3,124,376	Alaska	3,175,711	295,307
West Virginia	2,427,033	1,072,955	**Total**	403,084,127	127,113,884

### ResType classification results

A gradient-boosted model was trained on 70% of the labelled data and then tested on the remaining 30% of the labelled data. Table 6 show the performance (precision, recall, and F1-score) of the Region 1 ResType model on the test set. We used the macro average and weighted average to capture the metrics in class imbalances in the test set. The Support column shows the number of samples counted as Residential or Nonresidential in the final classification result.

**Table 6:** Region 1 Classification Results.

Class	Precision	Recall	F1-score	Support
Residential	92	95	93	82624
Nonresidential	94	92	93	82662

### Quality check of addresses

As an additional measure of quality control, we cross-referenced all city name, postal codes, and state pairings in the address source data with verified combinations of those data from authoritative sources including the US Postal Service, US Geologic Survey, US Census Bureau, Open Source data and HERE geospatial data^36–43^. We were able to create a comprehensive combination of city, state, and zip codes by validating through source data by capturing the geospatial relationships between the US Geological Survey National File^38^, the US Census Bureau Tiger Zip Code Tabulation Areas^40^, US Census Bureau Zip Code Tabulation Relationship File^39^, and the US Census Bureau Name Lookup Tables^41^. Pairings were validated by cross-referencing our new dataset against the US Postal Service Area and District, and Locale Detail^36,37^, resources and HERE geospatial data^43^. Address elements that were not found in these reference tables were excluded from the final processed address table as a verification and validation step.

## Usage Notes and Future Directions

There are several limitations of the current version of the datasets. The limitations mostly stem from the source data or imagery we used. While those limitations might be addressed in the future updates to the data, we advise the users having those in mind for specific applications.

### Limitations

Since these building outlines were derived from satellite imagery with a horizontal offset of up to 5 meters, these vectors will not align with all target imagery. Methods to shift building vectors to align with target imagery of choice will need to occur before performing additional analytics based on target imagery^44^. Another source of spatial misalignment is from the artifacts from the geometric simplification process. Even after carefully tuning shape regularization parameters, we have observed undesirable artifacts such as possible changes in structure geometries and location shifting. Further, these building outlines were derived from satellite imagery at a specific point in time. As such, temporal discrepancies may exist that could also result in structure changes in environment.

There are also several limitations regarding the addresses attribution. First, missing addresses in our structure data often reflect gaps in the availability of open source address data. Secondly, the steps we took to perform QA/QC checks, rank address records based on validity and completeness, and leverage ancillary datasets to guide the conflation process cannot compensate for poor data quality. For example, imprecise geolocations, such as those derived from linear referencing along street network centerlines, as well as invalid address elements, resulted in poor address conflation results in some areas. Some of these issues could be mitigated through improvements to our data cleaning and engineering methodology, but artifacts of these issues will be present in our data until the quality of the source data improves.

In addition, our address processing workflow and data cleaning procedures was primarily designed to process addresses that are typical for structures in the continental U.S. However, addresses found in the U.S. territories can be very different. While we took steps to adjust our address ranking process to account for some of these differences, further refinement is needed to more accurately capture addresses in those areas.

Currently, the process does not account for multi-address structures, such as townhouses, urban city blocks, and strip malls. Unlike many apartment buildings, which typically have a single street address with varying unit numbers, the aforementioned structures could have numerous street addresses with varying street numbers for a single contiguous structure. According to our approach, only one of those addresses would be captured for the structure. Future work would focus on a more comprehensive approach to account for multi-address structures.

### Future updates

We plan to work with the stakeholders and the funding agencies to provide updates to the datasets. The updates shall include the latest advances in computer vision for extracting information (i.e. structures) from recent high resolution remote sensing images, considerations of including other geospatial data modality, latest releases of source data that we used to populate critical attributions, and additional attributions that are useful to various applications.

## Data Availability

The sample selection process used ISOSCELES, a program written in Python 2.7 using the open source packages GDAL, OGR, SciPy, Numpy, Sci-kit Learn, and Pandas. It is available at https://github.com/btswan87/isosceles. Main geospatial data operations and manipulations use open packages, including Python, dask, sqalchemy2, geopandas, pandas, SciPy, Sci-kit Learn, psycopg2-binary, sqlalchemy, postgres, GDAL, OGR, DBeaver, and PostgreSQL. Regularization was performed using ArcPy. Database is a Docker image from CrunchyData with Postgres 14.2 and PostGIS 3.1.
